# Supplementary material for: A neuromorphic physiological signal processing system based on VO2 memristor for next-generation human-machine interface
Source: Nat Commun. 2023 Jun 21;14:3695. doi: 10.1038/s41467-023-39430-4 (PMC10284901; doi:10.1038/s41467-023-39430-4)
Supplement: Supplementary file 1 — Supplementary Information [file 41467_2023_39430_MOESM1_ESM.pdf]

Supplementary Information

A neuromorphic physiological signal processing system based on VO_2 memristor for next-generation human-machine interface

Rui Yuan[1,#], *Pek Jun Tiw*[1,#], *Lei Cai*[1], *Zhiyu Yang*[2], *Chang Liu*[1], *Teng Zhang*[1], *Chen Ge*[3], *Ru Huang*[1], *and Yuchao Yang*[1,2,4,5*]

[1]Beijing Advanced Innovation Center for Integrated Circuits, School of Integrated Circuits, Peking University, Beijing 100871, China

[2]School of Electronic and Computer Engineering, Peking University, Shenzhen 518055, China

[3]Beijing National Laboratory for Condensed Matter Physics, Institute of Physics, Chinese Academy of Sciences, Beijing 100190, China

[4]Center for Brain Inspired Chips, Institute for Artificial Intelligence, Frontiers Science Center for Nano-optoelectronics, Peking University, Beijing 100871, China

[5]Center for Brain Inspired Intelligence, Chinese Institute for Brain Research (CIBR), Beijing, Beijing 102206, China

[#]These authors contributed equally to the work.

E-mail: yuchaoyang@pku.edu.cn

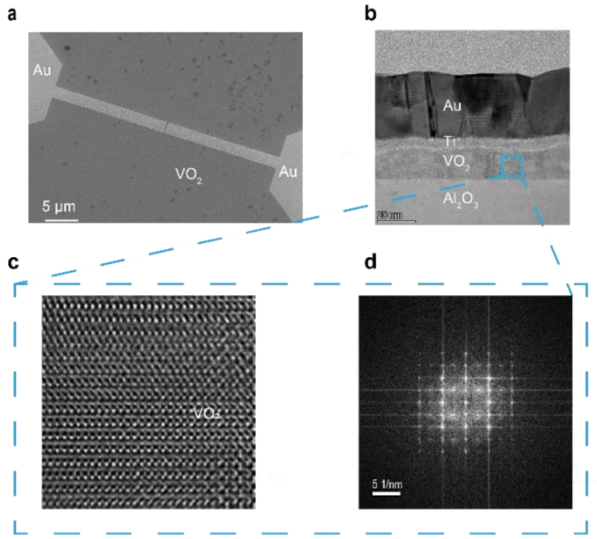

Supplementary Figure 1. Microstructure of VO_2 device. (a) Scanning electron microscopy (SEM) image of the VO_2 memristor. **(b)** Cross-sectional transmission electron microscopy (TEM) image of the VO_2 memristor. **(c)** Zoom-in views of the epitaxial VO_2 region. **(d)** The diffraction pattern extracted by fast Fourier transformation of **(c).**

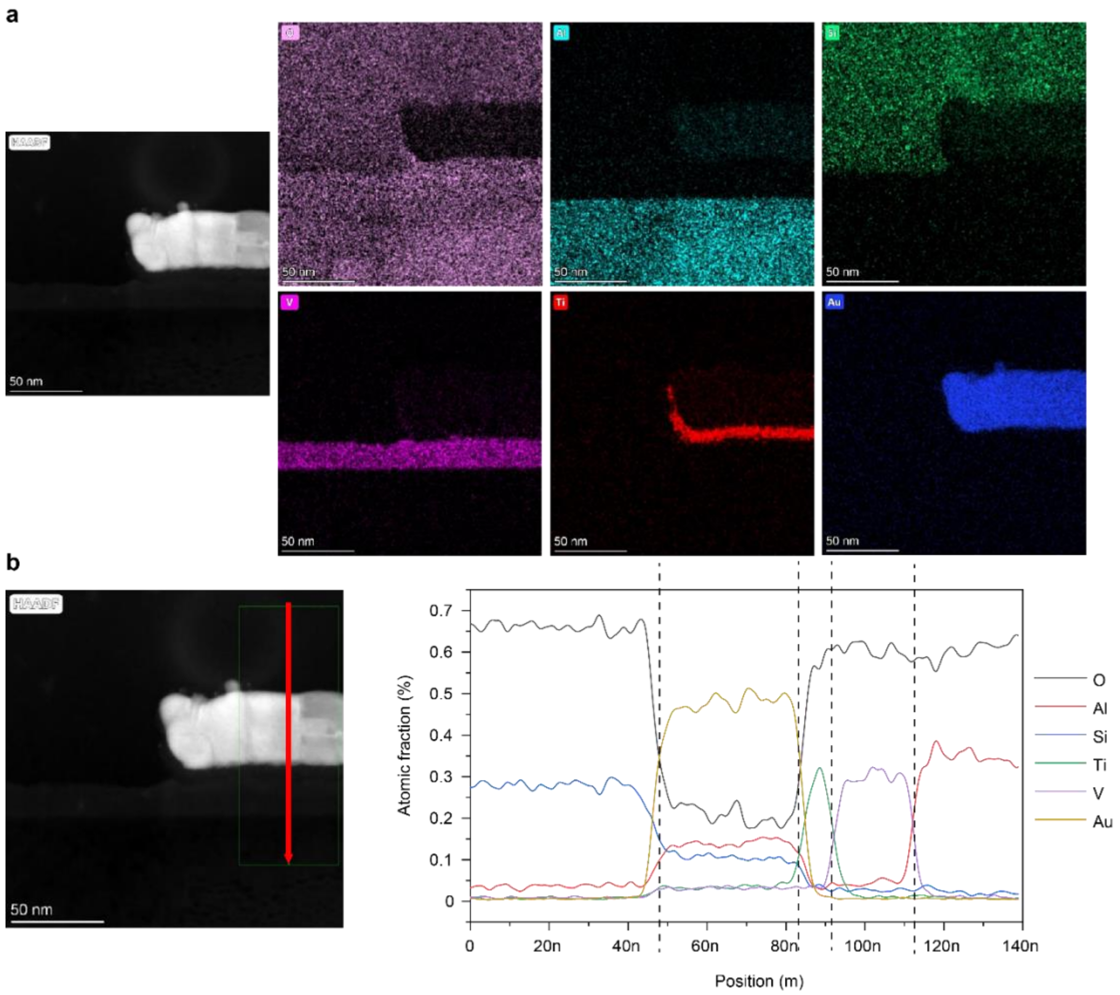

Supplementary Figure 2. The compositional characterization of VO_2 device. (a) Cross-sectional STEM image and corresponding EDS mapping of O, Al, Si, V, Ti and Au elements in the device. **(b)** EDS elemental line profile in the region of the device shown by the STEM image.

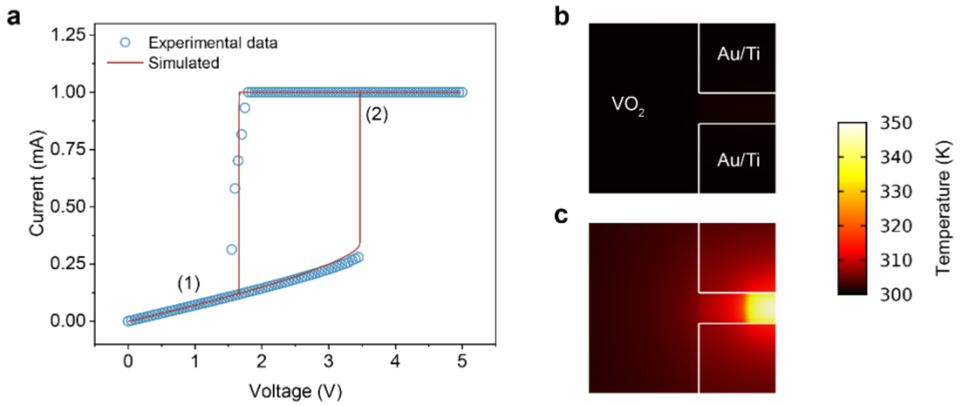

Supplementary Figure 3. COMSOL simulation results. (a) The simulated *I*-*V* curve agrees well with the experimental *I*-*V* curve. **(b)-(c)** Surface temperature maps of the VO_2 channel corresponding to points (1) and (2) on the *I*-*V* curve in (a), respectively. The switching of the device between HRS and LRS is accompanied by the formation or disappearance of a high-temperature metallic filament.

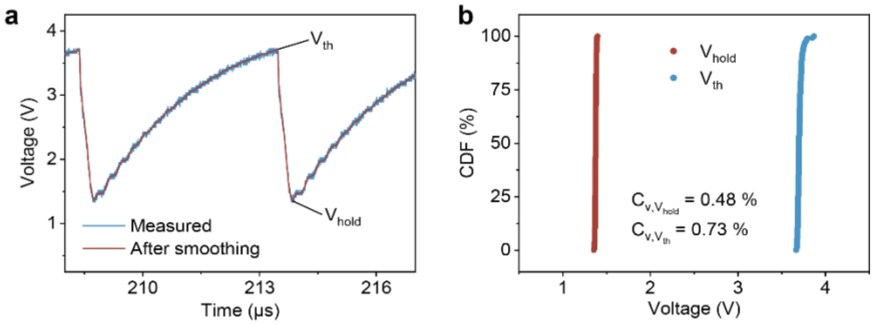

Supplementary Figure 4. V_{th} and V_{hold} variations under dynamical conditions. (a) The extraction of V_{th} and V_{hold} of a VO_2 memristor when it was connected to an external neuron circuit and allowed to oscillate. **(b)** The distribution of the extracted V_{th} and V_{hold} from ~1000 periods of oscillation illustrating very low variations.

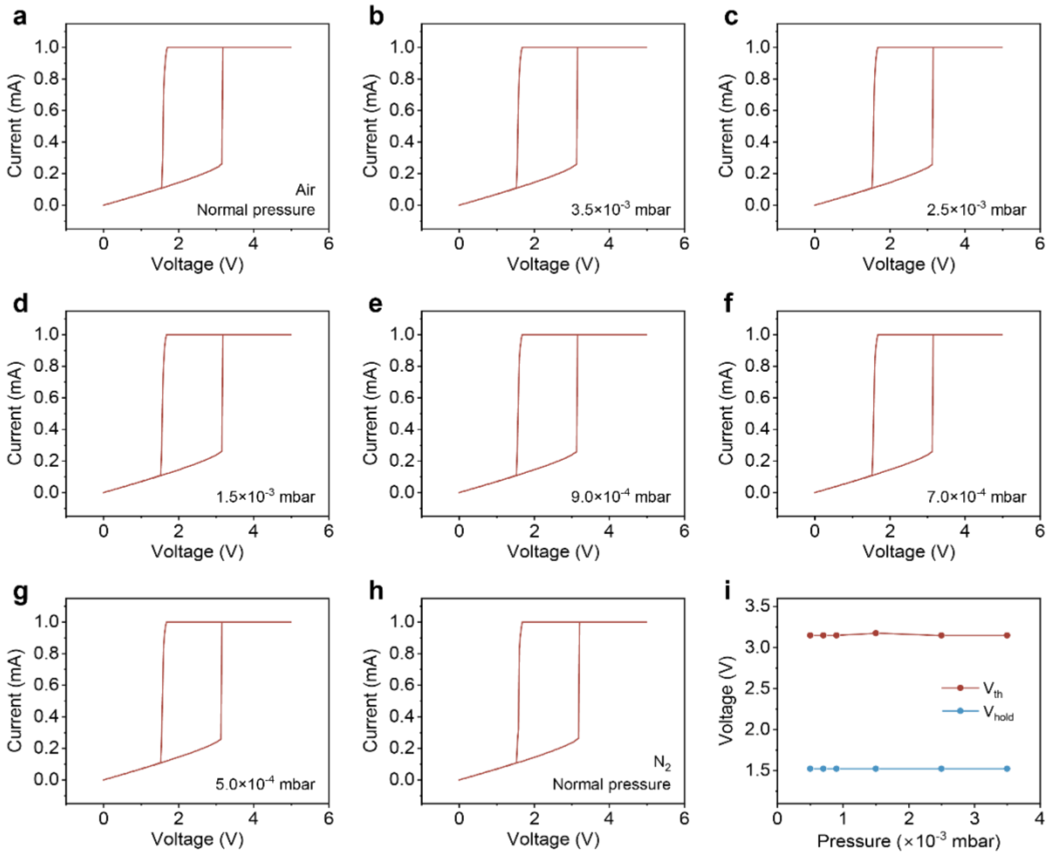

Supplementary Figure 5. Threshold switching behavior of the planar VO_2 device under different environments. The VO_2 device was operated **(a)** in air under normal atmospheric pressure, **(b)-(g)** under different ambient pressures ranging from 3.5×10^{-3} mbar down to 5.0×10^{-4} mbar and **(h)** in an N_2 environment. By doing so, the ambient moisture content was progressively reduced. No appreciable difference in the *I*-*V* curves can be observed. **(i)** The V_{th} and V_{hold} were also stable under different ambient pressures.

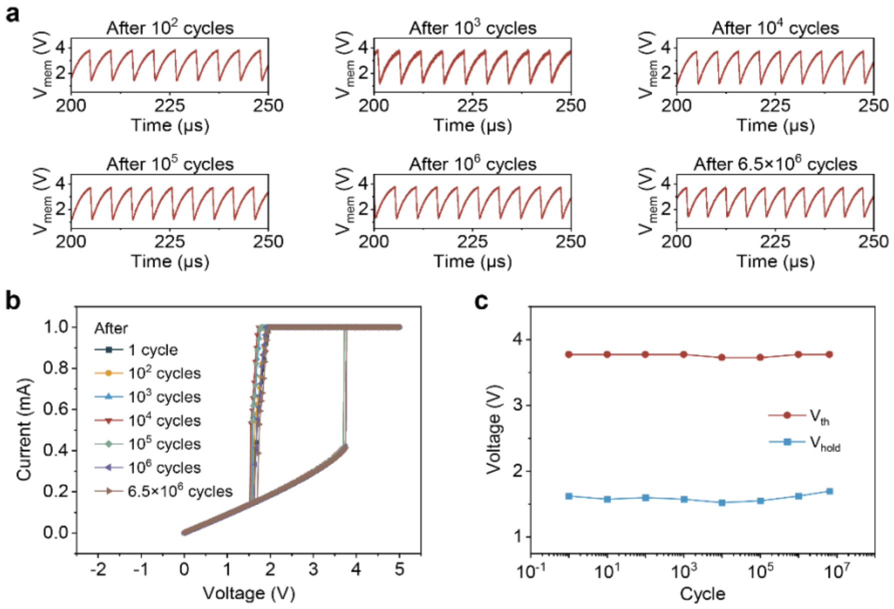

Supplementary Figure 6. Endurance of the VO_2 memristor. (a) Stable oscillating behavior of the VO_2 memristor circuit after 10^2, 10^3, 10^4, 10^5, 10^6 and 6.5×10^6 switching cycles. The endurance was tested by connecting the VO_2 memristor in a neuron circuit and repeatedly subjecting it to pulses with a width of 500 μs. **(b)** Stable *I*-*V* characteristics after 10^2, 10^3, 10^4, 10^5, 10^6 and 6.5×10^6 switching cycles. **(c)** V_{th} and V_{hold} extracted from the *I*-*V* characteristics.

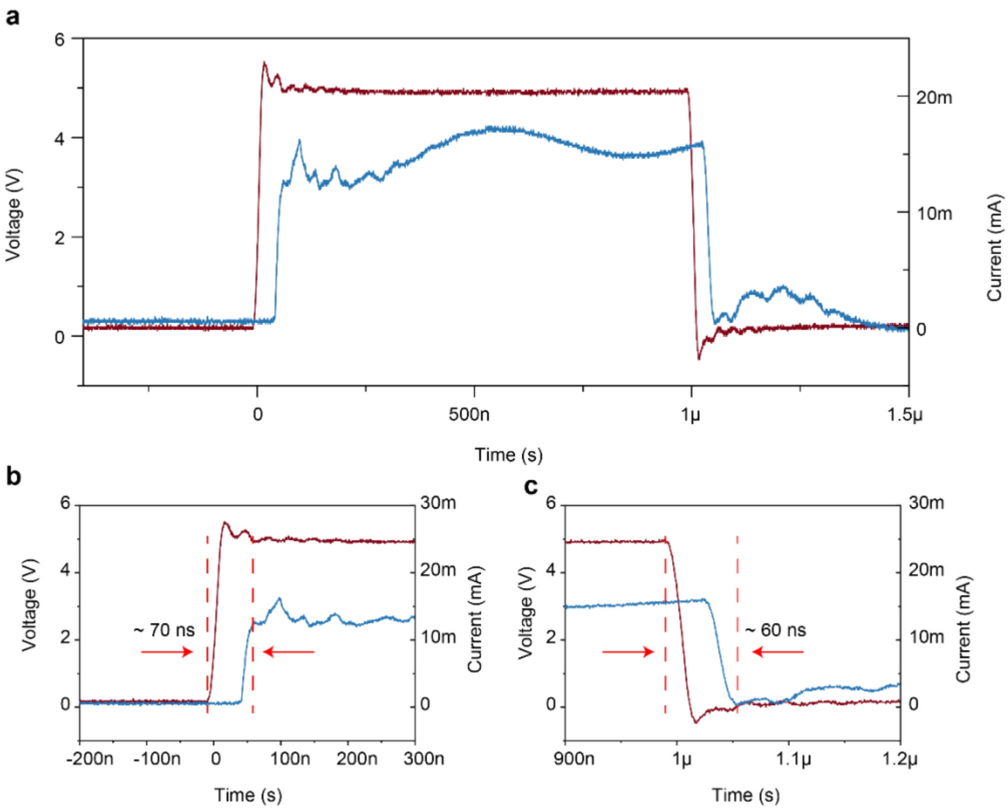

Supplementary Figure 7. Transient switching response of the VO_2 device. (a) Current waveform (blue curve) of the VO_2 device upon application of the voltage waveform (red curve). **(b)** The switching speed is <70 ns from off- to on-state. **(c)** The switching speed is <60 ns from on- to off-state.

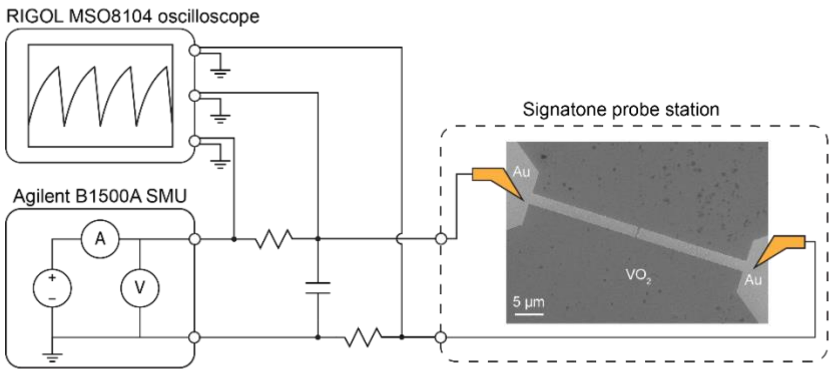

Supplementary Figure 8. Experimental setup to measure the behavior of the LIF neuron circuit. The VO_2 memristor was placed in a Signatone probe station to facilitate connections to the external circuit, which includes the Agilent B1500A source measurement unit and the RIGOL MSO8104 oscilloscope.

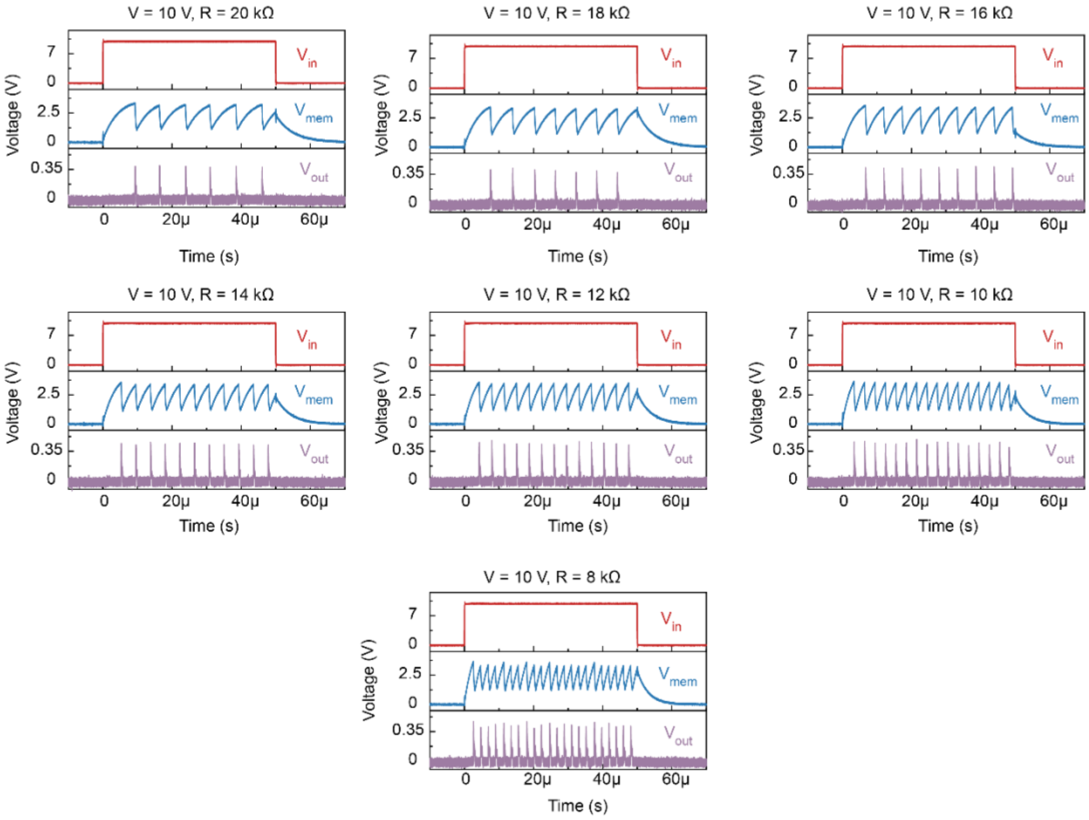

Supplementary Figure 9. The output results of spiking neuron using different series resistance. This figure shows the additional data under different series resistance R_L. When the series resistance increases, the input current will reduce, thus slowing down the charge process.

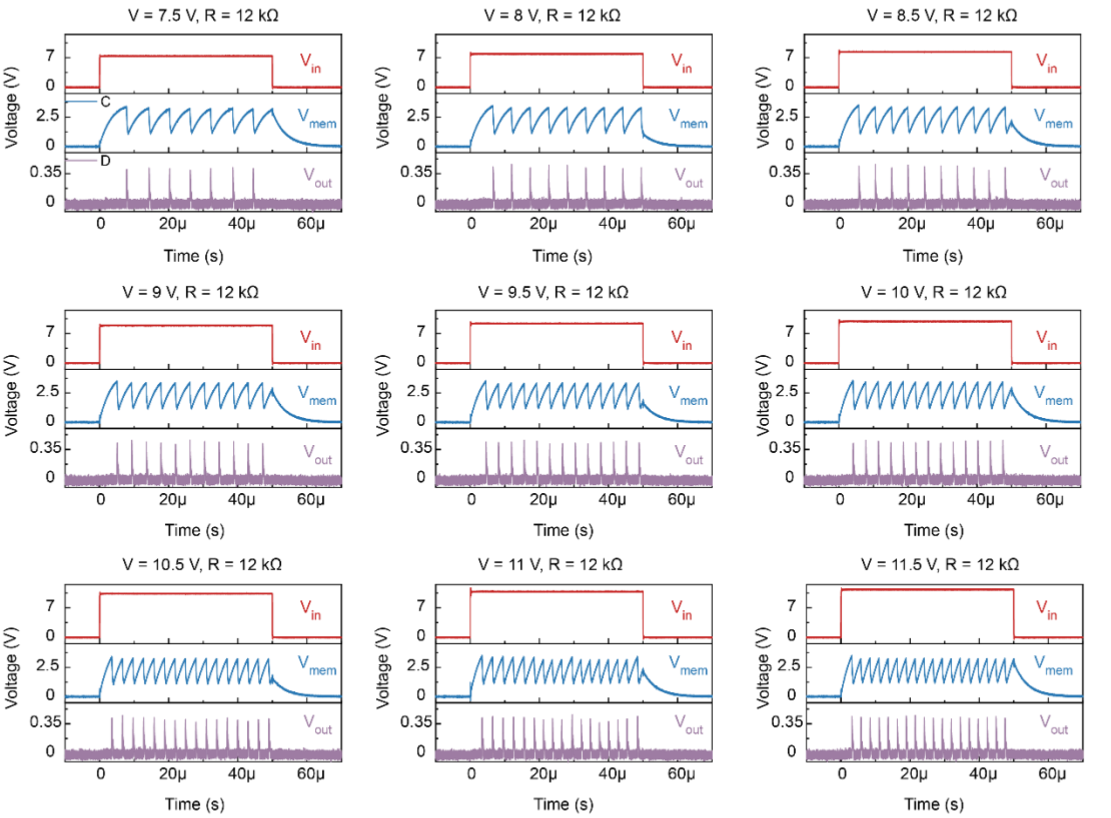

Supplementary Figure 10. The output results of spiking neuron under different applied voltage. This figure shows the additional data under different input voltages. larger input voltage will increase the charging current, thereby speeding up the frequency of spiking.

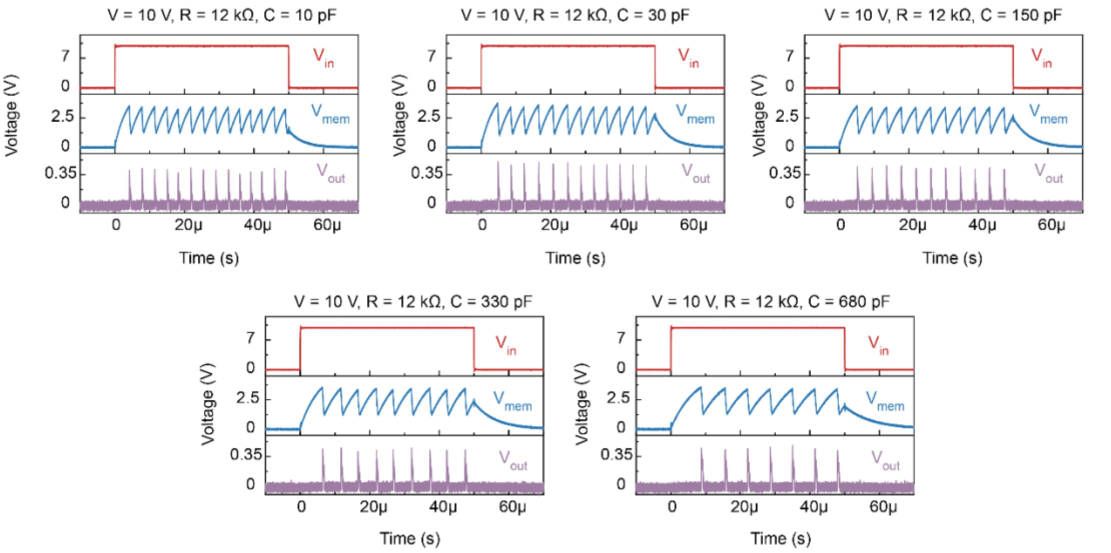

Supplementary Figure 11. The output results of spiking neuron under different parallel capacitors. This figure shows the additional data under different parallel capacitors.

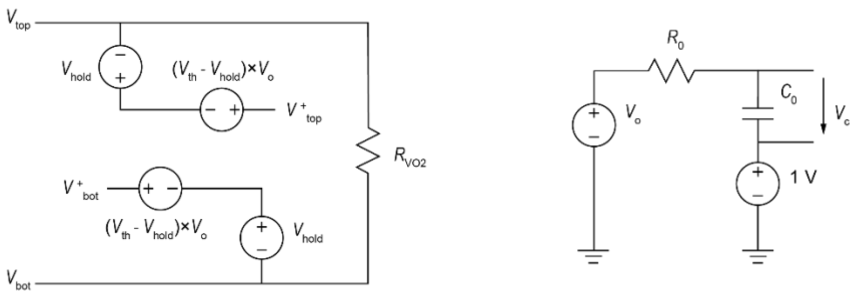

Supplementary Figure 12. The SPICE model of VO_2 memristor. The circuit on the left forms pairs of inputs for threshold comparison and sets the resistance of the memristor, while the circuit on the right determines the internal state of the model. R_{VO2} is the resistance of the memristor. V_{top} and V_{bot} represent the two terminal voltages of the memristor. V_{th} and V_{hold} are the threshold and holding voltages, respectively, of the memristor extracted from experimental data. V^+_{bot} and V^+_{top} are auxiliary voltage levels used by the comparator within the model. V_o represents the internal state (1 in HRS, 0 in LRS) given by the internal comparator output, which is modeled here using a behavioral voltage source. R_0 and C_0 form an RC circuit to model the finite resistive switching time. V_c is the voltage across C_0, which is used to determine R_{VO2}. The detailed workings of the model are given in the Method section of the main text.

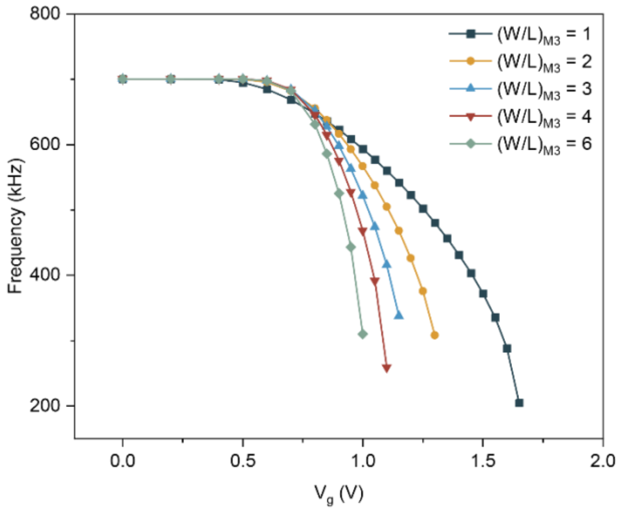

Supplementary Figure 13. Effect of V_g on the ALIF spiking frequency under different *W/L* ratios. The spiking frequency remains unchanged at V_g lower than the turn-on threshold voltage of transistor M_3 ($V_{t, M3}$). At V_g larger than $V_{t, M3}$, the spiking frequency decreases with increasing V_g. As V_g increases beyond a limit, the neuron ceases to fire. With a larger *W/L* ratio, the leakage current through M_3 is higher at a given V_g, resulting in a lower frequency and a lower V_g limit.

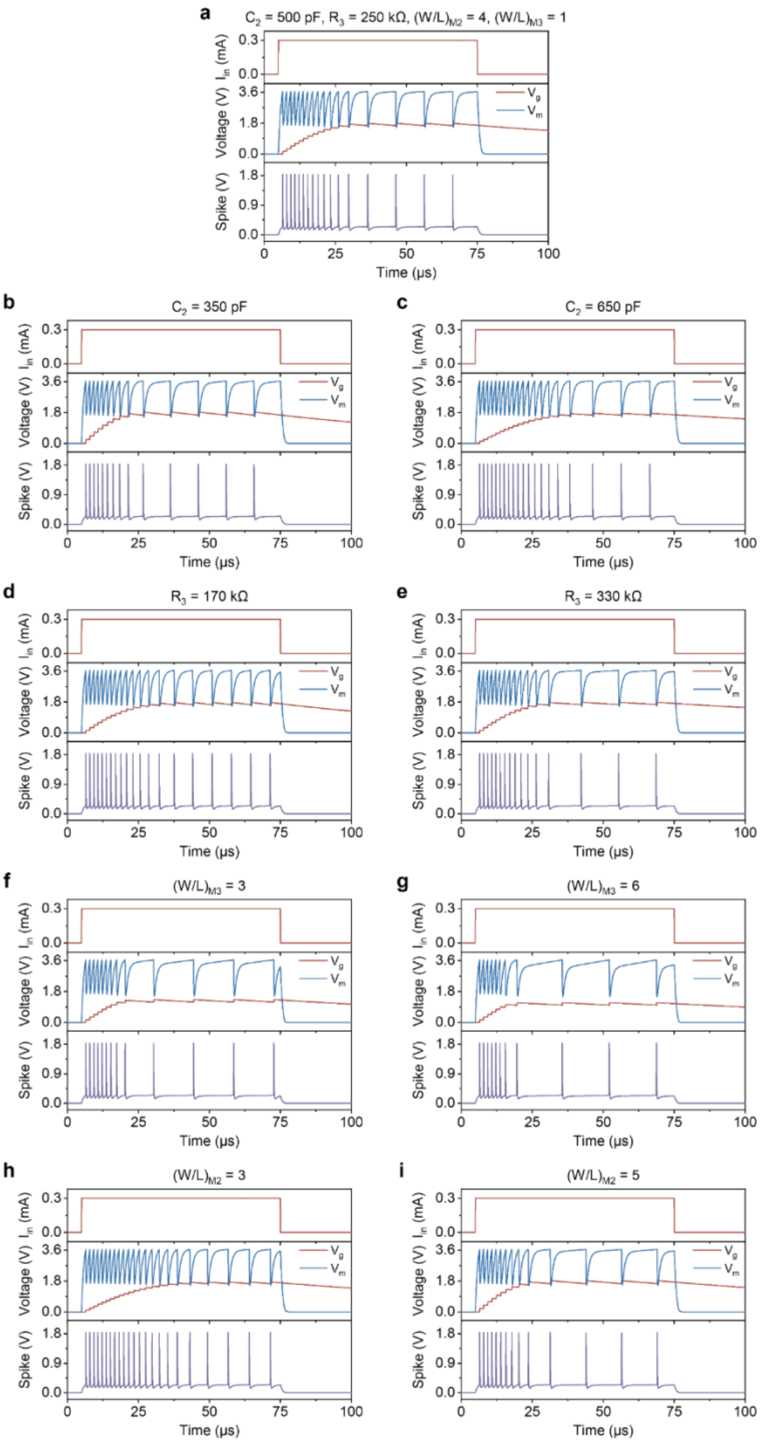

Supplementary Figure 14. Output of the ALIF neuron under different adaptive circuit parameters. Shown here are the responses of V_g, V_m, and V_{spike} to an input current pulse for **(a)** the control, **(b)-(c)** circuits with different C_2, **(d)-(e)** circuits with different R_3, **(f)-(g)** circuits with different M_3 W/L ratios, and **(h)-(i)** circuits with different M_2 W/L ratios. **(a)** is the same figure as in Fig. 3d of the main text, redrawn

here to enable comparison. The adaptive property is different under different conditions, suggesting that it can be specifically tuned by carefully adjusting these circuit parameters.

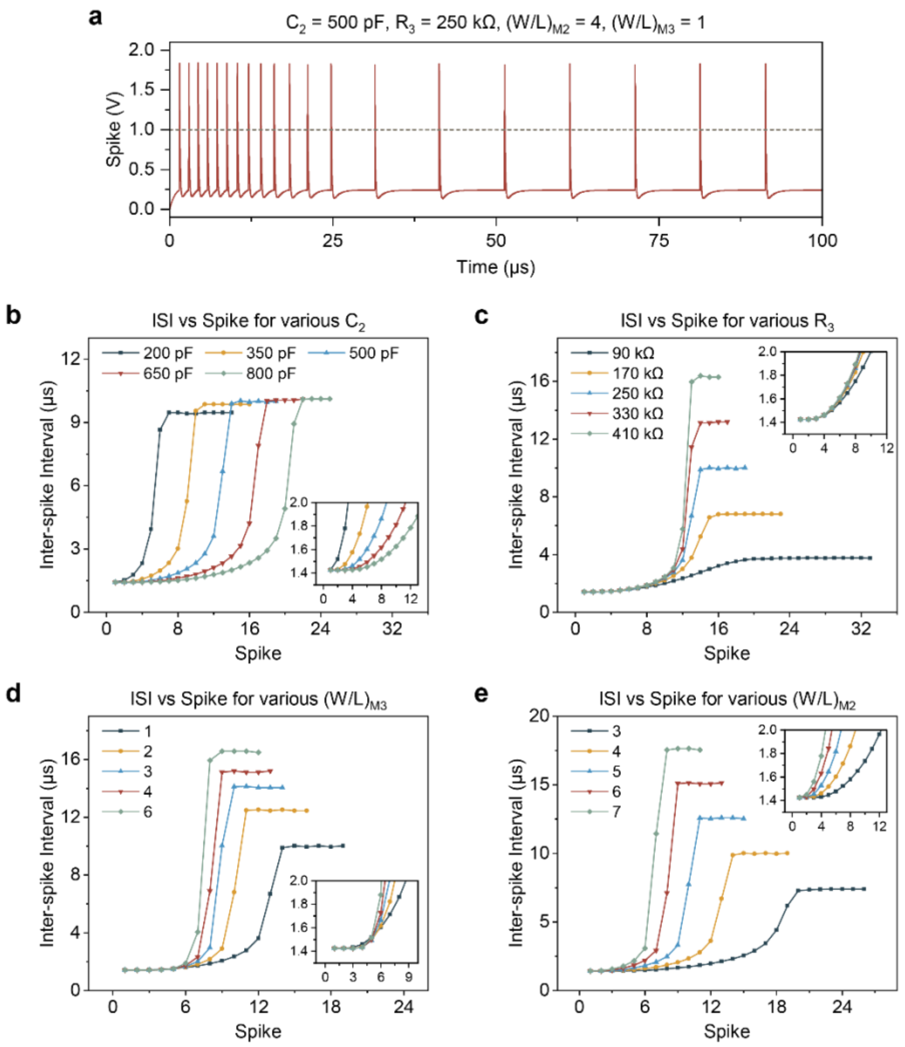

Supplementary Figure 15. Inter-spike intervals (*ISI*) of the ALIF neuron under different adaptive circuit parameters. (a) Spike output under controlled conditions (Supplementary Table 2). The dotted line is a guide used to determine the spike time, which is used to obtain *ISI*. **(b)-(e)** The evolution of *ISI* in terms of spike number under different **(b)** C_2, **(c)** R_3, **(d)** W/L of M_3, and **(e)** W/L of M_2. Insets are magnified plots showing the *ISI* during the initial few spikes. From the main plots, the saturation *ISI* can be tuned by R_3, W/L of M_2, and W/L of M_3. From the insets, the onset of adaptation can be tuned by C_2 and W/L of M_2. The rate of adaptation is modulated in all cases. All insets have the same abscissa and ordinate units as the main plots.

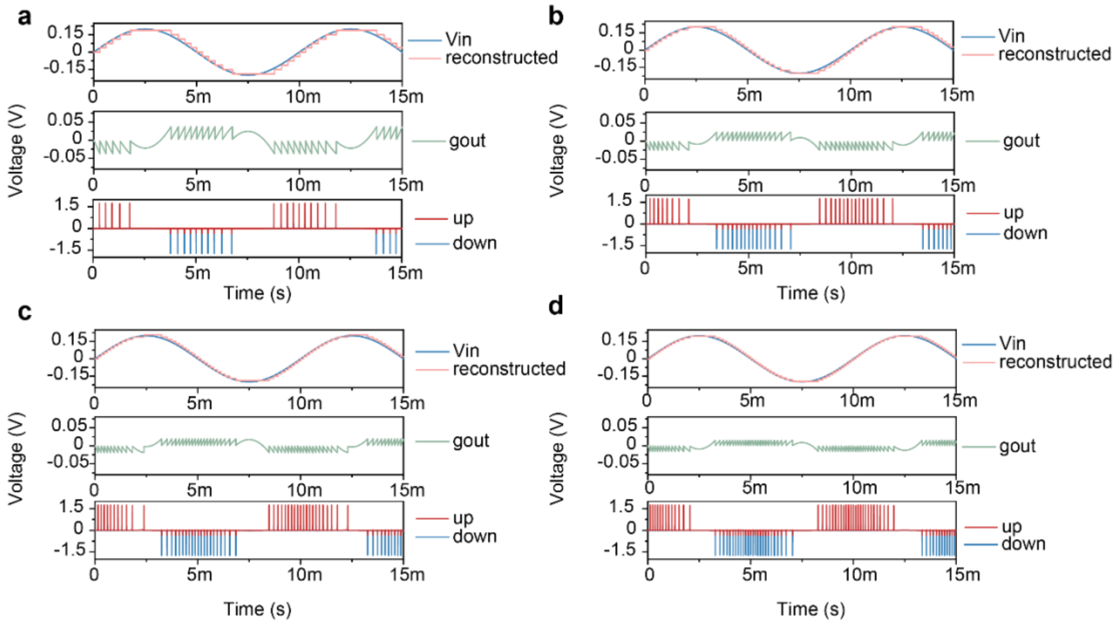

Supplementary Figure 16. The influence of amplification factor α of the intermediate stage op amp on the delta. The larger the α, the smaller the δ, the higher accuracy.

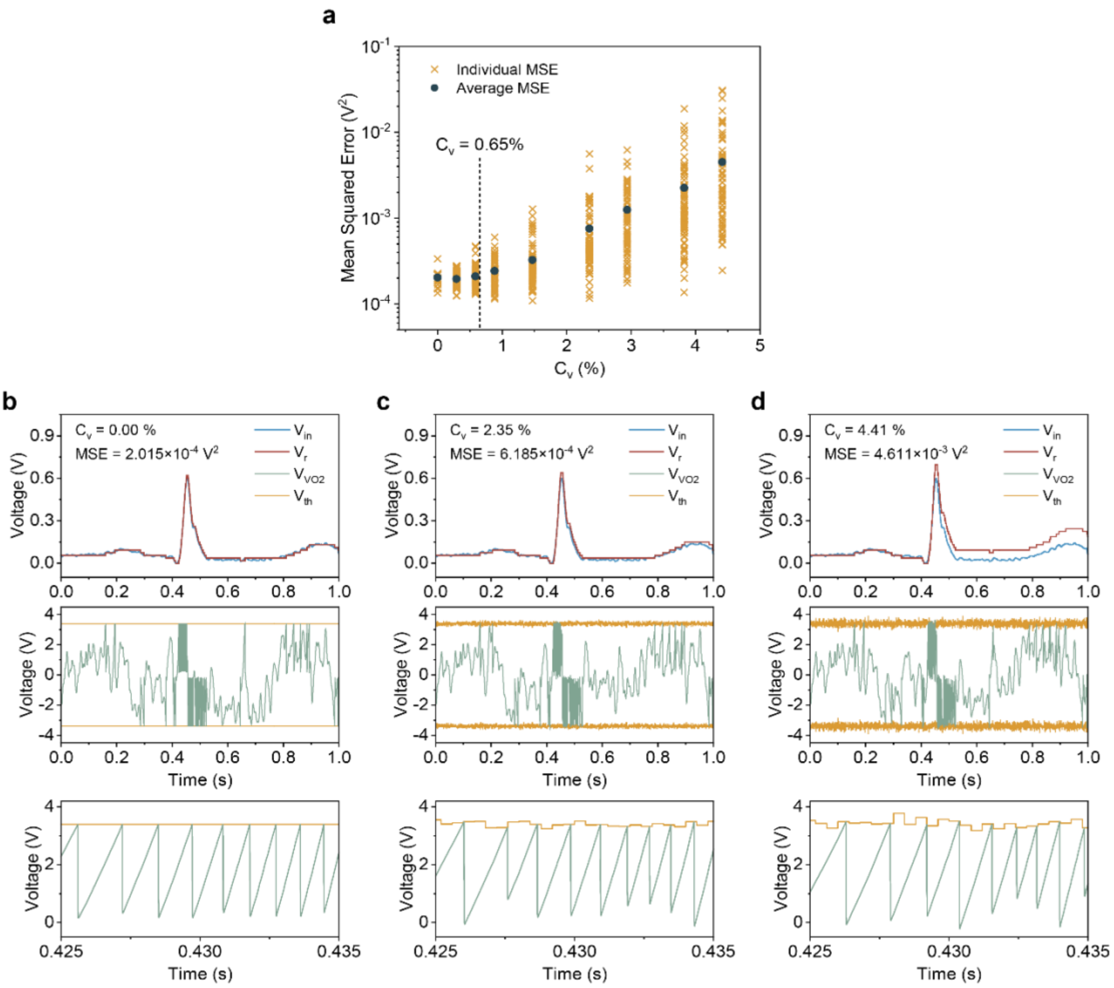

Supplementary Figure 17. The influence of C_v of V_{th} on the quality of signal encoding. (a) A scatter plot of the individual MSE between the original and the reconstructed signals for each of the 720 encoding trials and the average MSE for each C_v. The dotted line indicates the C_v of V_{th_pos} of our VO_2 memristor. **(b)-(d)** The original ECG signal (upper panel, blue), reconstructed signal (upper panel, red), voltage across the VO_2 memristor (middle and lower panel, green), and V_{th} (middle and lower panel, orange) under **(b)** zero, **(c)** moderate and **(d)** high degrees of fluctuations.

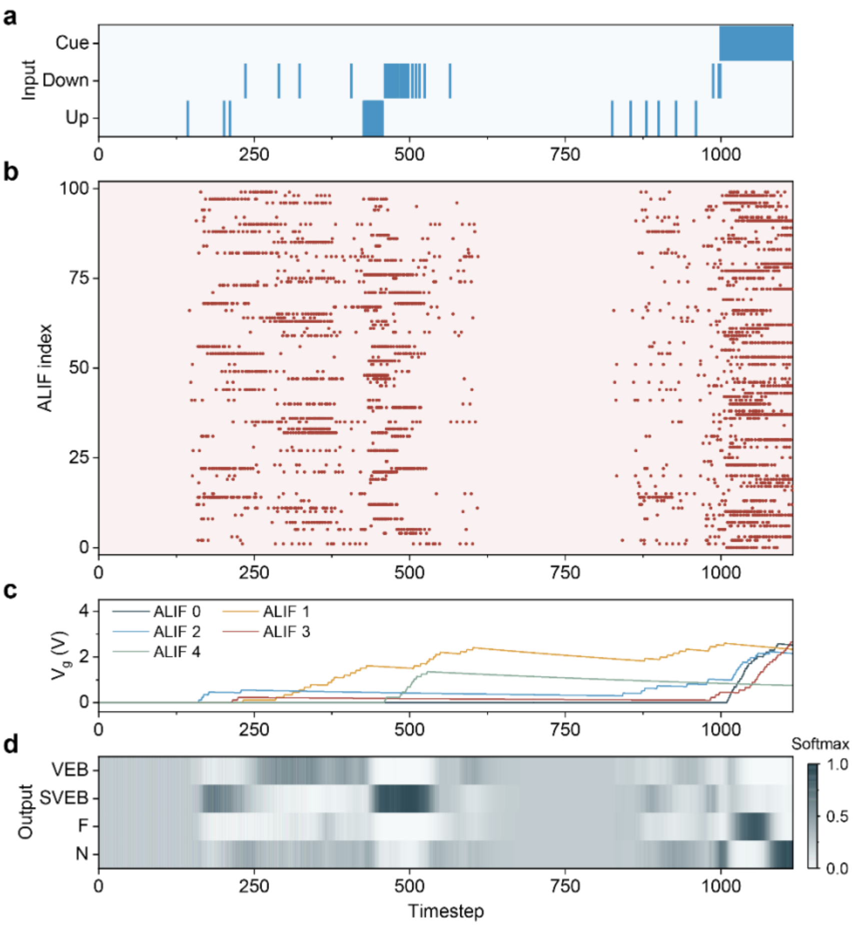

Supplementary Figure 18. The state evolution of an ALIF-only LSNN when classifying a normal heartbeat into one of four classes. (a) Input spike trains corresponding to the normal heartbeat shown in Fig. 5a of the main text. **(b)** Spike raster of the 100 ALIF neurons. **(c)** Evolution of V_g of five ALIF neurons. **(d)** Evolution of the output probabilities during classification.

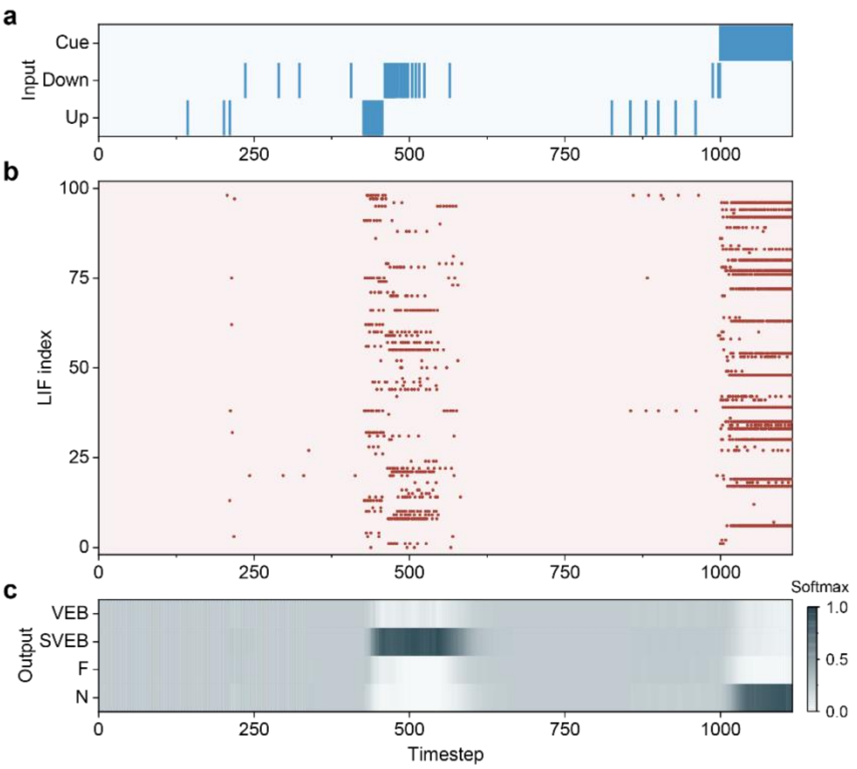

Supplementary Figure 19. The state evolution of a LIF-only LSNN when classifying a normal heartbeat into one of four classes. (a) Input spike trains corresponding to the normal heartbeat shown in Fig. 5a of the main text. **(b)** Spike raster of the 100 LIF neurons. **(c)** Evolution of the output probabilities during classification.

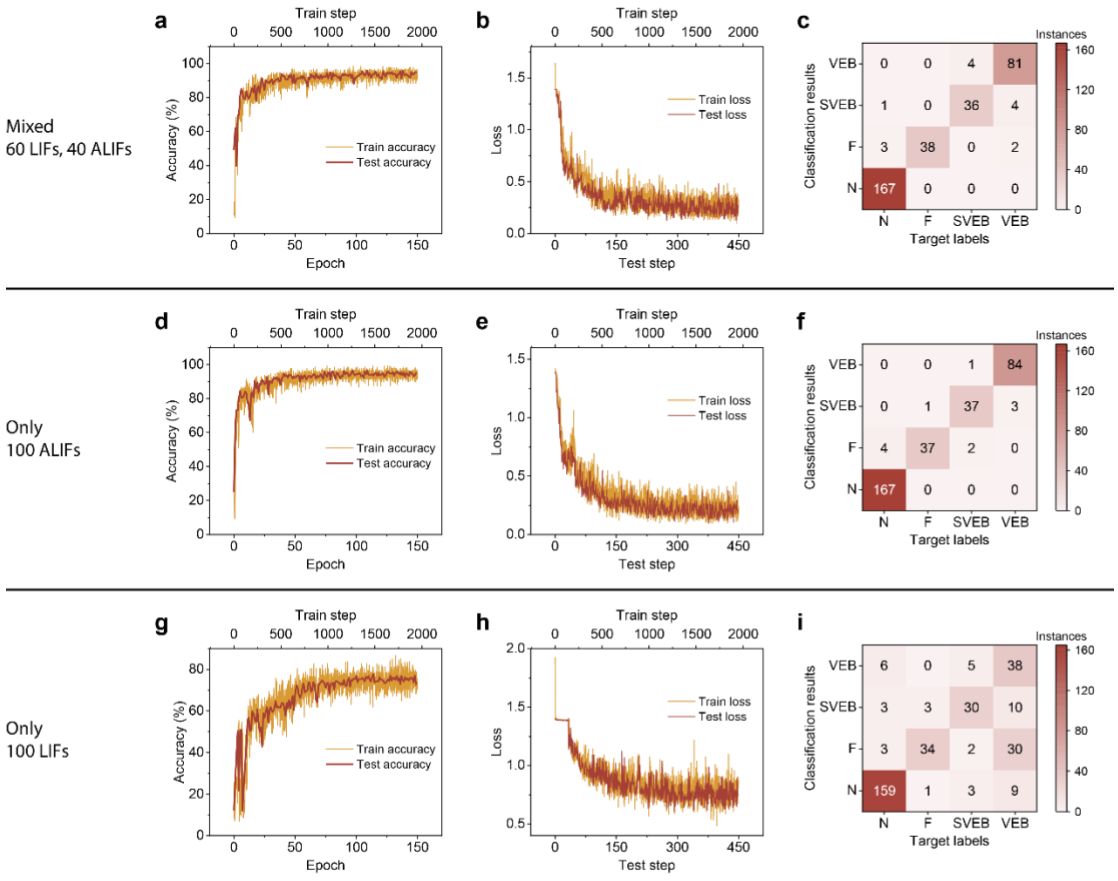

Supplementary Figure 20. A comparison of the performance of different LSNNs on the 4-class heartbeat classification task. (a) The evolution of the train and test accuracies, **(b)** the evolution of the train and test losses, and **(c)** the confusion matrix of the trained mixed LSNN with 60 hidden LIF neurons and 40 hidden ALIF neurons. **(d)-(f)** Similar plots for the ALIF-only LSNN for comparison. **(g)-(i)** Similar plots for the LIF-only LSNN for comparison. The mixed LSNN and ALIF-only LSNN each converged to a high accuracy and a low loss, while the LIF-only LSNN converged to a lower accuracy and higher loss.

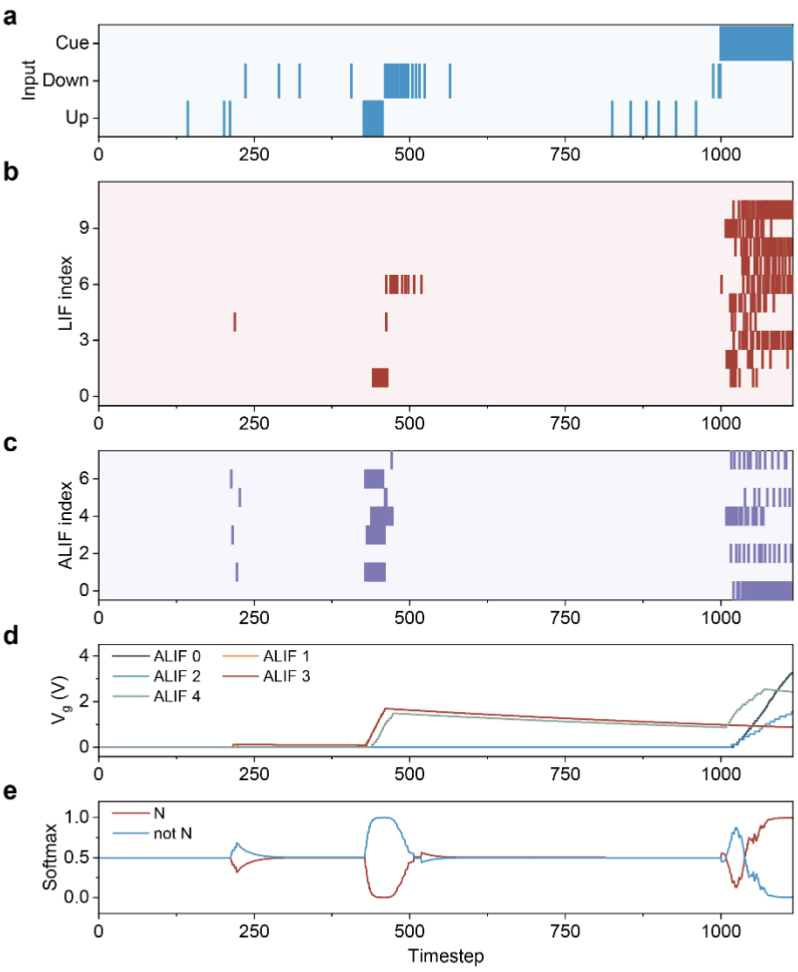

Supplementary Figure 21. The state evolution of a mixed LSNN when classifying a normal heartbeat into one of two classes. (a) Input spike trains corresponding to the normal heartbeat shown in Fig. 5a of the main text. **(b)** Spike raster of the 12 LIF neurons. **(c)** Spike raster of the 8 ALIF neurons. **(d)** Evolution of V_g of five ALIF neurons. **(e)** Evolution of the output probabilities during classification.

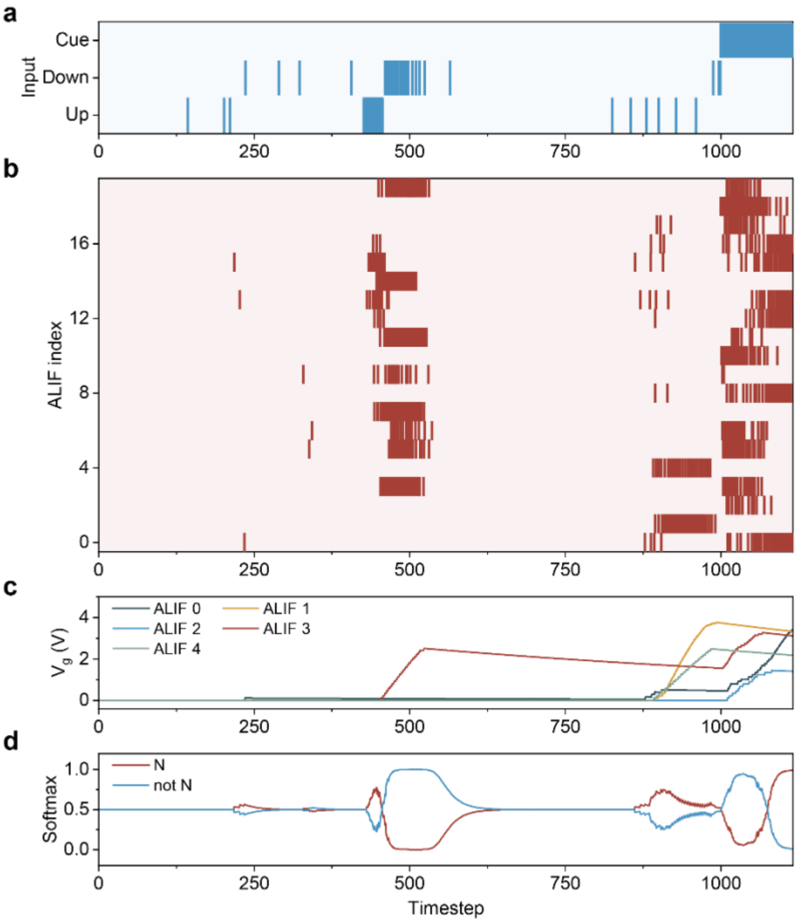

Supplementary Figure 22. The state evolution of an ALIF-only LSNN when classifying a normal heartbeat into one of two classes. (a) Input spike trains corresponding to the normal heartbeat shown in Fig. 5a of the main text. **(b)** Spike raster of the 20 ALIF neurons. **(c)** Evolution of V_g of five ALIF neurons. **(d)** Evolution of the output probabilities during classification.

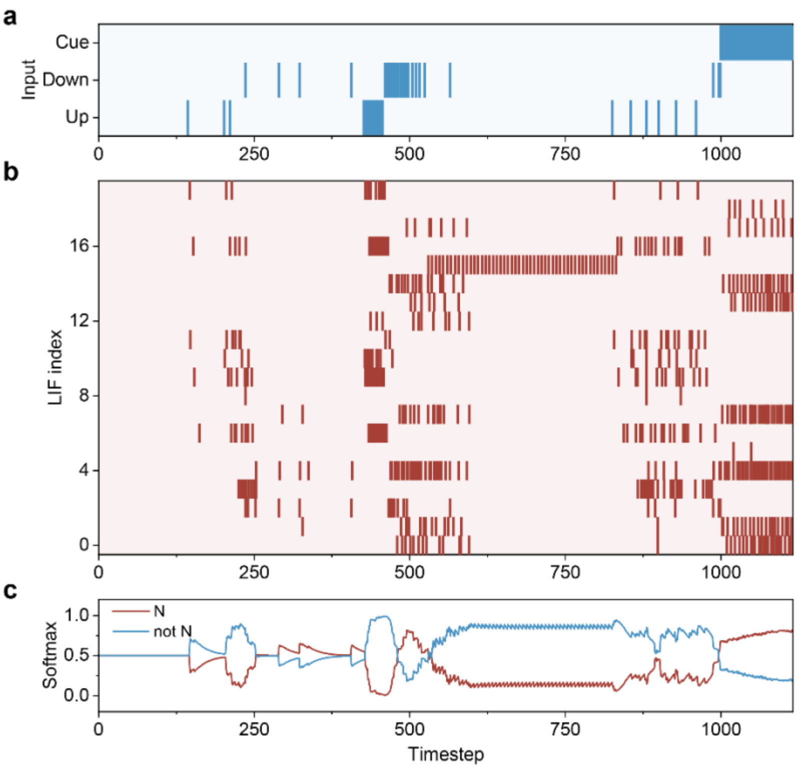

Supplementary Figure 23. The state evolution of a LIF-only LSNN when classifying a normal heartbeat into one of two classes. (a) Input spike trains corresponding to the normal heartbeat shown in Fig. 5a of the main text. **(b)** Spike raster of the 20 LIF neurons. **(c)** Evolution of the output probabilities during classification.

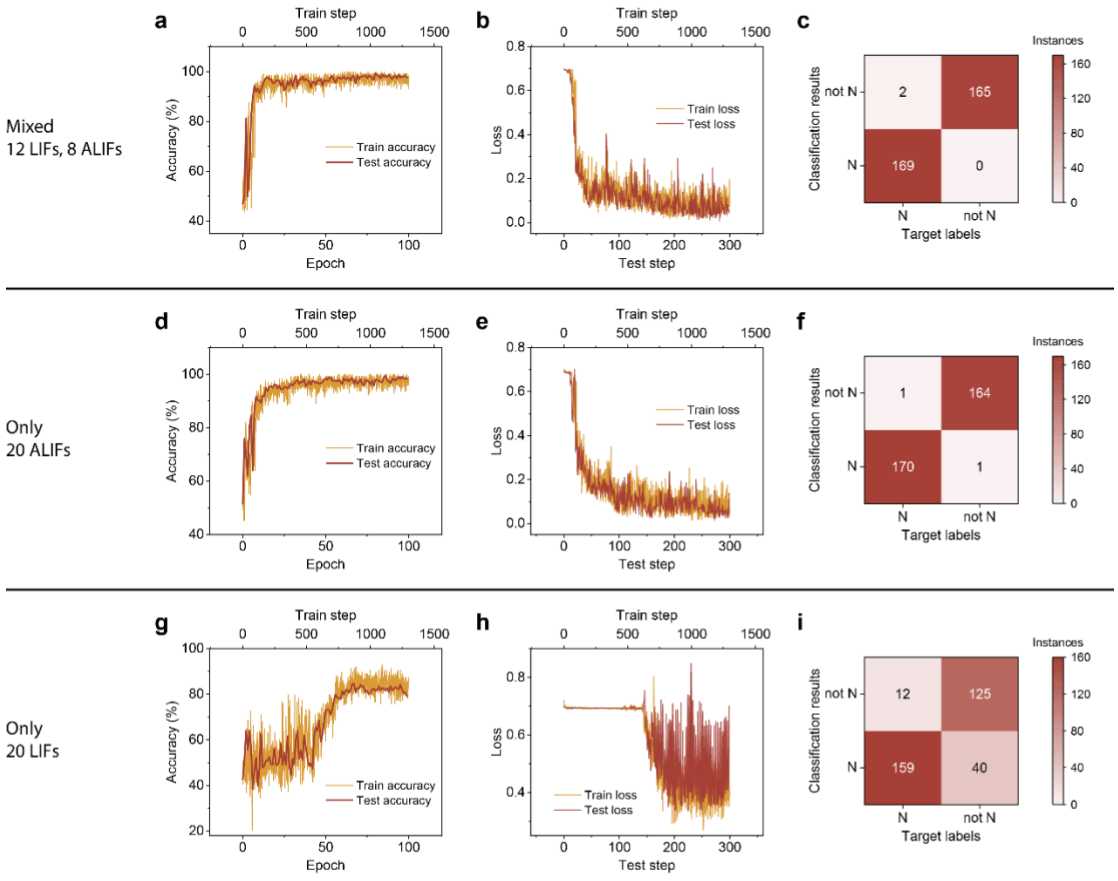

Supplementary Figure 24. A comparison of the performance of different LSNNs on the 2-class heartbeat classification task. (a) The evolution of the train and test accuracies, **(b)** the evolution of the train and test losses, and **(c)** the confusion matrix of the trained mixed LSNN with 12 hidden LIF neurons and 8 hidden ALIF neurons. **(d)-(f)** Similar plots for the ALIF-only LSNN for comparison. **(g)-(i)** Similar plots for the LIF-only LSNN for comparison. The mixed LSNN and ALIF-only LSNN each converged to a high accuracy and a low loss, while the LIF-only LSNN converged to a lower accuracy and higher loss. The consistently low accuracies in the first half of the training epochs in **(g)** and the highly fluctuating losses in **(h)** indicate that it is difficult for the small-sized LIF-only LSNN to learn the task.

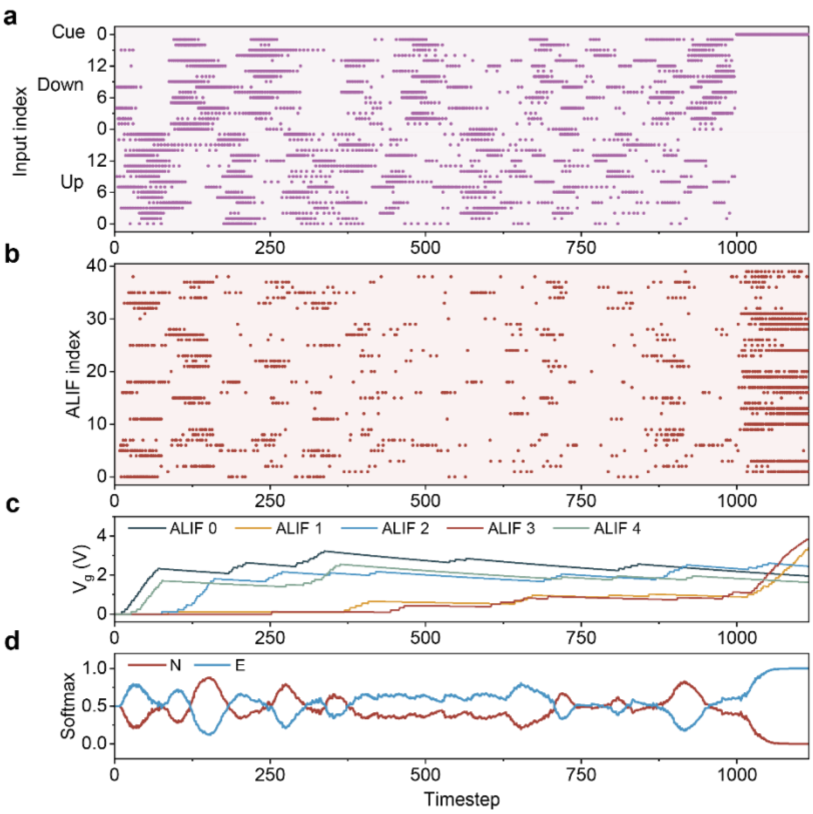

Supplementary Figure 25. The state evolution of an ALIF-only LSNN when classifying an epileptic EEG clip. (a) Input spike trains corresponding to the epileptic EEG clip shown in Fig. 6a of the main text. **(b)** Spike raster of the 40 ALIF neurons. **(c)** Evolution of V_g of five ALIF neurons. **(d)** Evolution of the output probabilities during classification.

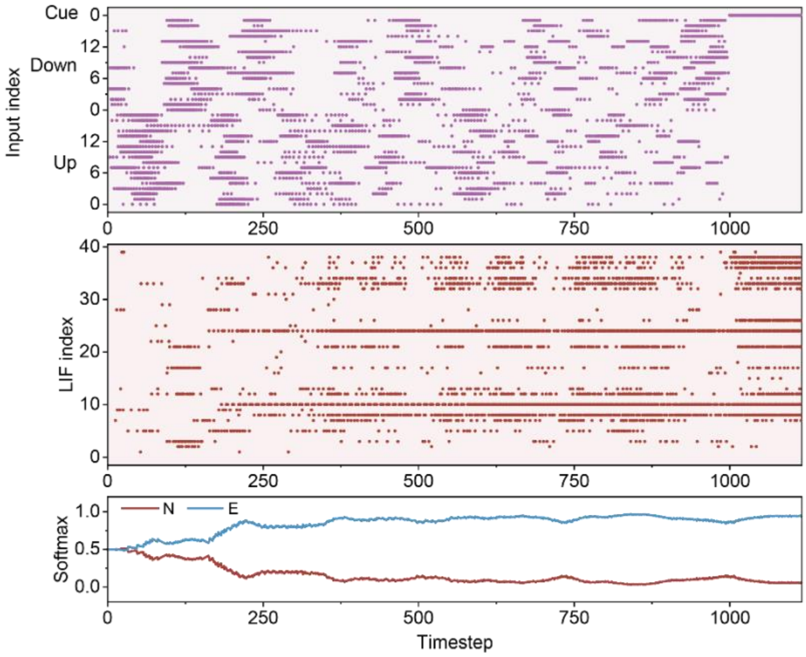

Supplementary Figure 26. The state evolution of a LIF-only LSNN when classifying an epileptic EEG clip. (a) Input spike trains corresponding to the epileptic EEG clip shown in Fig. 6a of the main text. **(b)** Spike raster of the 40 LIF neurons. **(c)** Evolution of the output probabilities during classification.

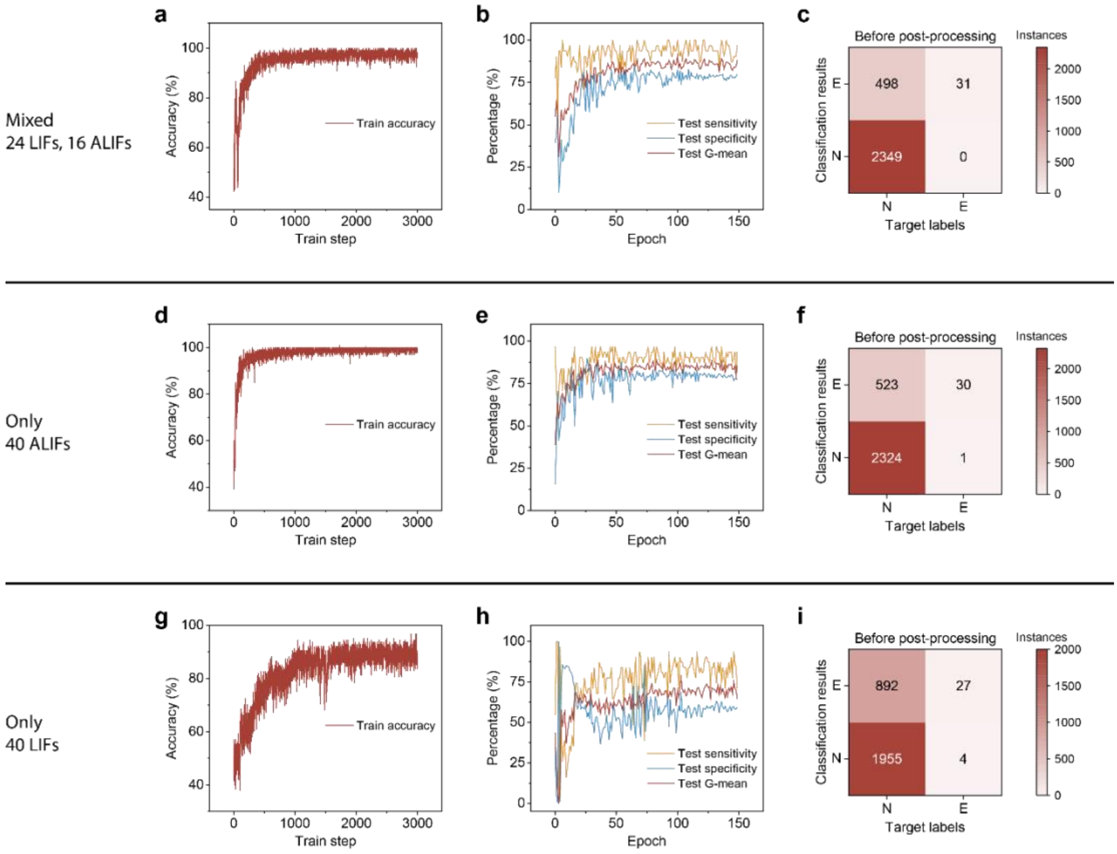

Supplementary Figure 27. A comparison of the performance of different LSNNs on the epileptic seizure detection task. (a) The evolution of the train accuracy, **(b)** the evolution of the test sensitivity, specificity and G-mean, and **(c)** the confusion matrix of the trained mixed LSNN with 24 hidden LIF neurons and 16 hidden ALIF neurons. **(d)-(f)** Similar plots for the ALIF-only LSNN for comparison. **(g)-(i)** Similar plots for the LIF-only LSNN for comparison. Both the mixed LSNN and ALIF-only LSNN performed better than the LIF-only LSNN in terms of the training and testing metrics presented here.

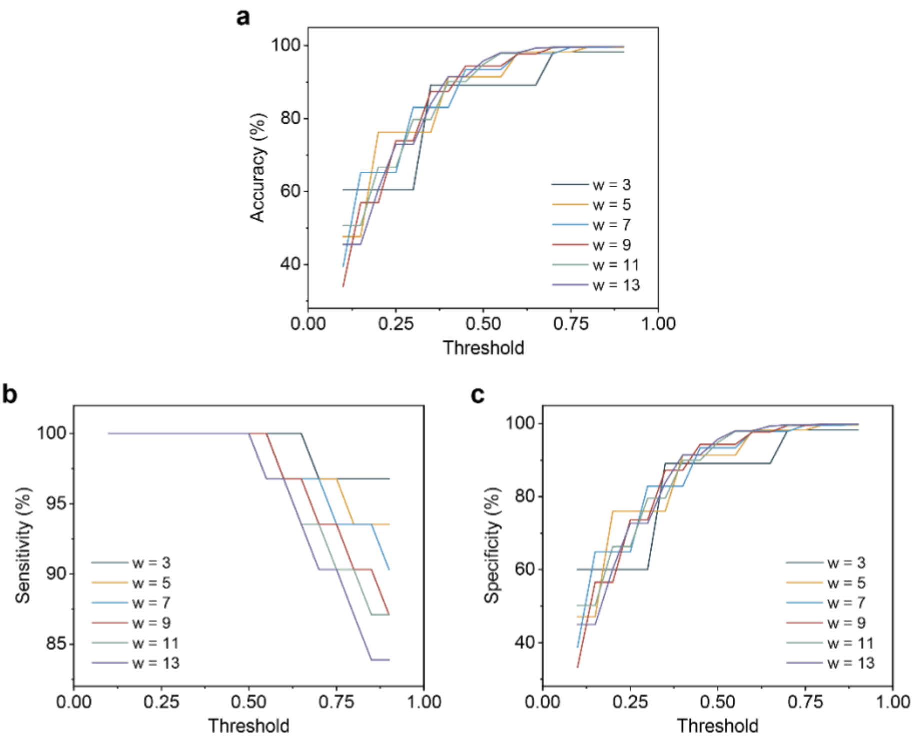

Supplementary Figure 28. The effect of moving average window width and threshold on the post-processing performance. The effect of threshold on the post-processing **(a)** accuracy, **(b)** sensitivity, and **(c)** specificity under different moving average window width. As can be seen, there exists a trade-off between sensitivity and specificity when selecting a threshold value. Increasing the threshold will reduce the possibility of classifying true negatives (lower moving average values) as false positives, but will, above a certain point, increase the possibility of classifying true positives (higher moving average values) as false negatives. As the test dataset has much more negative samples (N) relative to positive samples (E), the accuracy versus threshold curves closely follows the corresponding specificity versus threshold curves.

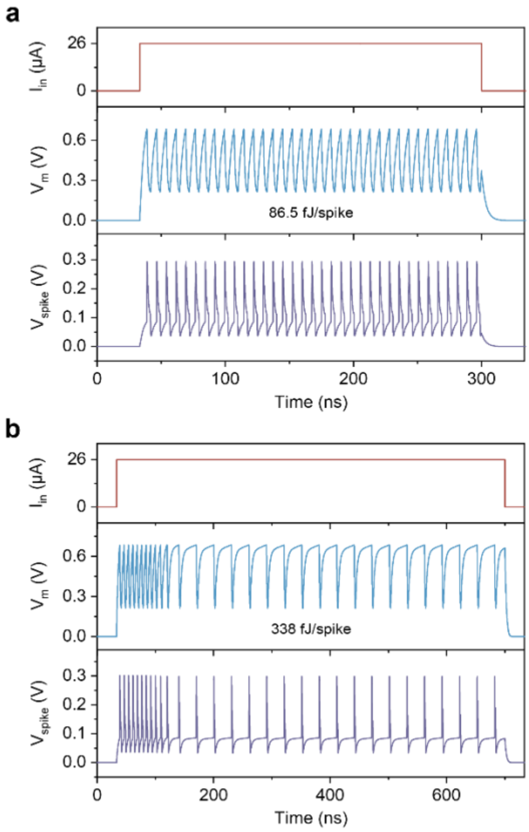

Supplementary Figure 29. Energy consumption of the optimized LIF and ALIF neurons. (a) Energy per spike of the optimized LIF neuron obtained by integrating the transient power ($I_{in}\times V_m$) over time and then dividing the result by the number of spikes. **(b)** Energy per spike of the optimized ALIF neuron obtained using the same procedure, with the transient power being the sum of $I_{in}\times V_m$ and the power drawn by the adaptive control circuit ($I_{Vdd}\times V_{dd}$).

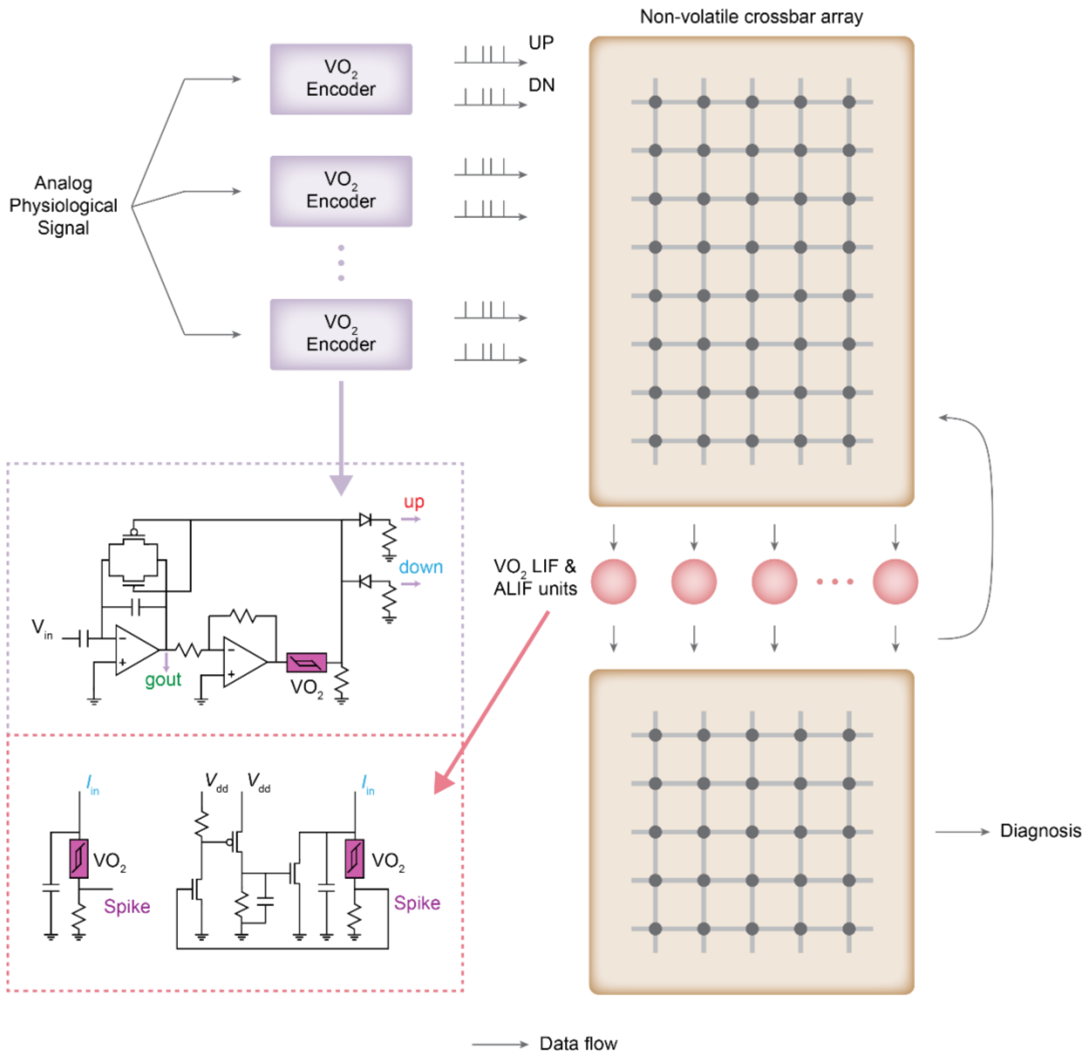

Supplementary Figure 30. An overview of a compact hardware-based spiking neural network. To achieve high-density integration, such a network may employ our VO_2 memristor-based asynchronous spike encoder to convert analog signals into spikes, as well as VO_2 memristor-based LIF and ALIF neurons to perform biologically plausible neural computations. Further area savings can be made by utilizing dense non-volatile crossbar arrays of emerging memories for synaptic computations.

Supplementary Table 1. Parameters of device

Parameter	Value	Units
V_{th}	3.4	V
V_{hold}	1.45	V
R_{on}	150	Ω
R_{off}	14000	Ω
R_0	1	Ω
C_0	100	nF
α	5000	-

Supplementary Table 2. Parameters of ALIF

V_{dd}	5 V
I_{in}	0.3 mA
R_1	1 kΩ
R_2	500 kΩ
R_3	250 kΩ
C_1	60 pF
C_2	500 pF
M_1	L = 0.5 µm, W = 15 µm
M_2	L = 0.5 µm, W = 2 µm
M_3	L = 0.5 µm, W = 0.5 µm

Supplementary Table 3. Benchmark of adaptive LIF circuit

	Neuron circuit
Indiveri et al. [1]	22 FETs + 1 capacitor
Indiveri[2]	17 FETs + 3 capacitors
Ferreira et al. [3]	10 FETs + 3 capacitors + 1 resistor
Ahmadi-Farsani et al. [4]	24 FETs + 3 capacitors
Wang et al. [5]	6 FETs + 1 capacitor + 1 resistor + 2 memristors+1 amplifier
Shaban et al. [6]	6 FETs + 1 resistor + 1 memristor+1 integrator + 1 comparator + 1 pulse generator
This work	3 FETs + 2 capacitors + 3 resistors + 1 memristor

Supplementary Table 4. Benchmark of spike encoder

		Sharifshazileh[7]	He[8]	This work
Bulky modules	Controller	-	1	-
	DAC	-	1	-
	Buffer	2	-	-
	Charge adder	-	2	-
	Comparator	2	2	-
	Amplifier	1	1	2
Discrete components	Transistor	9	-	2
	Capacitor	3	-	2
	Diode	-	-	2
	Resistor	-	-	5
	Memristor	-	-	1

Supplementary Table 5. Parameters of spike encoder

Electric source	V_{dd} = 5 V, V_{ss} = -5 V
M_1	W = 0.5 µm, L = 12.5 µm
M_2	W = 0.5 µm, L = 15 µm
C_1	50 pF
C_2	50 pF
R_1	100 Ω
R_2	20 kΩ
R_3	1.5 kΩ
R_4	5 kΩ
R_5	5 kΩ

Supplementary Table 6. Parameters of LSNN

	ECG	EEG	Units
V_{dd}	5	5	V
V_{th_eff}	3.6	3.6	V
V_{hold_eff}	1.5	1.5	V
R_{eff}	15.5	15.5	kΩ
R_3	100	100	kΩ
C_1	0.7168	1.613	μF
C_2	5.556	12.5	μF
$(W/L)_{M2}$	8	8	-
$(W/L)_{M3}$	16	16	-
Δt	0.5556	1.25	ms
$\tau_{low\text{-}pass}$	20	20	dt
Max synaptic delay	10	10	dt
I_{in} scaling	0.005	0.005	-
λ_f	10^{-7}	10^{-7}	-
f_0	10	10	Hz
γ	0.3	0.3	-

Supplementary Table 7. Statistics of the best test accuracy (%) in each of 18 runs

Run	2-class ECG			4-class ECG		
	12 LIF<br>8 ALIF	20 LIF	20 ALIF	60 LIF<br>40 ALIF	100 LIF	100 ALIF
1	96.73	78.27	97.62	94.05	74.11	94.94
2	95.24	67.86	99.40	95.24	70.24	94.05
3	99.40	70.54	98.81	92.86	75.30	94.94
4	97.62	76.79	98.51	92.56	73.51	95.54
5	98.21	73.81	97.92	90.48	72.32	95.54
6	97.62	76.49	98.51	91.96	76.19	91.96
7	95.83	72.02	98.21	89.29	75.60	96.43
8	97.02	69.64	98.81	89.58	75.89	94.64
9	98.81	76.79	98.51	94.64	73.81	96.73
10	97.92	75.00	98.81	94.35	72.62	94.64
11	97.92	80.36	97.92	88.39	73.51	95.83
12	98.21	83.93	90.18	95.83	68.15	91.37
13	97.92	84.52	93.45	94.35	75.60	93.75
14	97.92	80.65	99.11	93.15	70.83	95.54
15	98.51	68.75	97.32	90.77	73.51	94.64
16	93.15	80.95	95.83	91.37	74.11	92.86
17	97.32	80.95	98.51	91.07	72.02	95.83
18	98.21	79.46	96.73	89.88	77.68	89.88
Average	97.42	76.49	97.45	92.21	73.61	94.40
σ	1.458	5.146	2.297	2.233	2.349	1.827

Supplementary Table 8. Statistics of the best test G-mean (%) in each of 18 runs

Run	EEG		
	24 LIF 16 ALIF	40 LIF	40 ALIF
1	90.83	70.35	88.88
2	85.91	69.70	83.79
3	86.44	77.34	80.47
4	86.87	66.39	80.31
5	79.68	67.05	82.32
6	84.94	70.00	85.64
7	81.09	73.98	81.33
8	85.19	71.10	82.88
9	89.25	72.48	83.84
10	83.87	74.64	76.11
11	88.96	70.06	77.68
12	87.07	72.44	78.09
13	83.58	70.00	78.14
14	85.96	69.46	73.67
15	85.55	74.40	81.80
16	86.26	68.50	82.87
17	88.55	71.52	83.76
18	87.30	68.48	86.80
Average	85.96	70.99	81.58
σ	2.759	2.821	3.837

Supplementary Table 9. Benchmark of network

	Year	Dataset	Dataset Processing	Weight number	Sensitivity (%)	Specificity (%)	Accuracy (%)
Liang et al. [9]	2019	CHB-MIT	2D array (time * channels) + 2D-CNN-LSTM	9695012	84.00	99.00	99.00
Boonyakitanont et al. [10]	2019	CHB-MIT	DWT, feature extraction, normalization + 1D-CNN	73584	66.76	99.63	99.07
Wei et al. [11]	2019	CHB-MIT	MIDS, WGANs + 1D-CNN	210272	72.11	95.89	84.00
Wang et al. [12]	2021	CHB-MIT	2D array (time * channels), RS-DA strategy + S-1D-CNN	133698	88.14	99.62	99.54
This Work	2022	CHB-MIT	Spike-encoding, LSNN, post process	3160	90.32	99.89	99.79

Supplementary Table 10. Parameters of the optimized ALIF

V_{dd}	1.2 V
I_{in}	26 μA
R_1	4 kΩ
R_2 (PMOS current source as load)	L = 260 nm, W = 65 nm, $V_{g,bias}$ = 0.6 V
R_3 (NMOS current source as load)	L = 1040 nm, W = 65 nm, $V_{g,bias}$ = 0.4 V
C_1	100 fF
C_2	30 fF
M_1	L = 65 nm, W = 65 nm
M_2	L = 100 nm, W = 100 nm
M_3	L = 65 nm, W = 65 nm

Supplementary Table 11. Detailed comparison between adaptive LIF circuits

	Area (μm^2)	Energy per spike (J)	Adapted frequency (Hz)	FOM (Hz μm^{-2} J^{-1})	Notes
Indiveri et al. (2006) [1]	2573	900×10^{-12}	-	-	• CMOS-based
Indiveri (2007) [2]	-	-	-	-	• CMOS-based
Ferreira et al. (2019) [3]	120	3.6×10^{-15}	205×10^{3}	4.75×10^{17}	• CMOS-based
Ahmadi-Farsani et al. (2022) [4]	863	140×10^{-12}	1×10^{3}	8.28×10^{9}	• CMOS-based
Wang et al. (2018) [5]	~320	-	30×10^{6}	-	• Memristor-based<br>• Area estimated by calculating capacitor area only and using a capacitance density of 2.5 fF μm^{-2}.
Shaban et al. (2021) [6]	11435.7	158×10^{-12}	-	-	• Memristor-based<br>• Energy based on reported values for TSMC 65 nm ASIC simulation and spike count for SMNIST.
This work	~224000	6.92×10^{-9}	100×10^{3}	6.45×10^{7}	• Memristor-based<br>• Unoptimized.<br>• Area estimated using a capacitance density of 2.5 fF μm^{-2}.
	~53.4	338×10^{-15}	33.2×10^{6}	1.84×10^{18}	• Memristor-based<br>• Optimized.<br>• VO_2 based on ref[13].<br>• Circuit based on PTM 65 nm MOSFETs.<br>• Area estimated using a capacitance density of 2.5 fF μm^{-2} and a sheet resistance of 690 Ω □$^{-1}$.

Supplementary Table 12. Parameters of the optimized spike encoder

Electric source	V_{dd} = 1.2 V, V_{ss} = -1.2 V
M_1	W = 23 μm, L = 2.5 μm
M_2	W = 13 μm, L = 2.5 μm
D_1 (Diode-connected PMOS)	W = 1.3 μm, L = 65 nm
D_2 (Diode-connected NMOS)	W = 0.7 μm, L = 65 nm
C_1	800 fF
C_2	800 fF
R_1	500 Ω
R_2	16.5 kΩ
R_3	1 kΩ
R_4	100 kΩ
R_5	100 kΩ

Supplementary Table 13. Detailed comparison between spike encoders

	Area (μm^2)	Notes
Sharifshazileh et al. (2021) [7]	~13000	• CMOS-based<br>• Area estimated from chip design layout provided
He et al. (2021) [8]	~17618	• CMOS-based<br>• Area estimated from chip photograph provided
This work	~2231	• Memristor-based<br>• Optimized.<br>• VO_2 based on ref[13].<br>• Op-amp based on ref[14].<br>• Circuit based on PTM 65 nm MOSFETs.<br>• Area estimated using a capacitance density of 2.5 fF μm^{-2} and a sheet resistance of 690 Ω $\square^{-1}$.

Supplementary Note 1: The rationale behind choosing a planar structure for the VO_2 memristor

The main reason for using planar VO_2 devices is due to their outstanding cycle-to-cycle (C2C) uniformity. To understand how the planar structure allows for high uniformity, we must first consider the origin of device variations. The stochasticity of VO_2 stems from the existence of multiple domains in the film and the competition between these domains in nucleating and initiating filament formation[15, 16], which means that stochasticity can be effectively suppressed by obtaining high crystallinity VO_2 films. High crystallinity epitaxial VO_2 can be grown on *c*-Al_2O_3 via pulsed laser deposition as evident from Supplementary Fig. 1 and is attributed to the matching lattice planes across the film-substrate interface[17]. The fabrication of planar VO_2 devices only requires the deposition of electrodes after film growth, which does not compromise the excellent characteristics of the film-substrate interface and the film itself, therefore enabling low C2C variations as can be seen from Fig. 2b-c. To quantify such variations, we computed the coefficient of variation, which is defined by $C_v = \sigma/\mu$. The C_v of V_{th_pos}, V_{hold_pos}, V_{th_neg}, and V_{hold_neg} were 0.65%, 0.86%, 0.31% and 1.68%, respectively. In addition to the uniformity observed under steady state, the VO_2 memristor also displayed very small variations in V_{th} and V_{hold} when it was connected to an external circuit and was operating in a dynamical state (Supplementary Fig. 4). The C_v of V_{th} and V_{hold} during transient oscillations were 0.73% and 0.48%, respectively. On the contrary, vertical VO_2 devices necessitate growing VO_2 on a bottom electrode and often result in polycrystalline or non-stoichiometric amorphous films[18, 19], of which the latter requires additional post-annealing or electroforming procedures to form a polycrystalline channel. Therefore, the C2C variation in vertical devices is often larger than in epitaxial planar devices.

The importance of C2C uniformity in our system is further exemplified by the results shown in Supplementary Fig. 17, wherein the impact of C2C variations in the form of V_{th} fluctuations on the encoding ability of the VO_2 memristor-based asynchronous spike encoder was studied (see also Supplementary Note 3 for details). Larger C2C variations in V_{th} was found to be detrimental to the encoding quality. Therefore, we opted for the more superior planar structure instead of the vertical structure to ensure that physiological signal processing systems incorporating VO_2 devices can function reliably.

Supplementary Note 2: An RC circuit analysis of the LIF neuron

The artificial LIF neuron is essentially an RC circuit. Starting from the voltage differential equation, the time evolution of the capacitor voltage can be derived and the charging time t_r from V_{hold} to V_{th} can then be expressed by Eq. 1:

$$t_{\mathrm{r}} = [R_{\mathrm{L}} \parallel (R_{\mathrm{off}}+R_0)]C_{\mathrm{p}} \cdot \ln\left(\frac{\frac{R_{\mathrm{off}}+R_0}{R_{\mathrm{L}}+R_{\mathrm{off}}+R_0}V_{\mathrm{in}} - V_{\mathrm{hold}}}{\frac{R_{\mathrm{off}}+R_0}{R_{\mathrm{L}}+R_{\mathrm{off}}+R_0}V_{\mathrm{in}} - V_{\mathrm{th}}}\right) \quad (1)$$

R_{on} and R_{off} denote the resistance of the LRS and HRS, respectively. Similarly, the discharging time t_f from V_{th} to V_{hold} can be described by Eq. 2:

$$t_{\mathrm{f}} = [R_{\mathrm{L}} \parallel (R_{\mathrm{on}}+R_0)]C_{\mathrm{p}} \cdot \ln\left(\frac{V_{\mathrm{th}} - \frac{R_{\mathrm{on}}+R_0}{R_{\mathrm{L}}+R_{\mathrm{on}}+R_0}V_{\mathrm{in}}}{V_{\mathrm{hold}} - \frac{R_{\mathrm{on}}+R_0}{R_{\mathrm{L}}+R_{\mathrm{on}}+R_0}V_{\mathrm{in}}}\right) \quad (2)$$

Thus, the frequency can be calculated by Eq. 3:

$$f = \frac{1}{t_{\mathrm{r}} + t_{\mathrm{f}}} \quad (3)$$

As t_r dominates the spiking period, the frequency is then approximately equal to the inverse of t_r. Subsequently, a brief analysis of the effect of circuit parameters on the frequency can be made. Observe that the natural logarithmic term is a decreasing function of the inner voltage dividing term, which in turn increases with V_{in} and decreases with R_L. Besides, the term $R_L \parallel (R_{off} + R_0)$ decreases as R_L decreases. Thus, a smaller R_L or a larger V_{in} decreases t_r and increases the frequency. Furthermore, t_r is a linear function of C_p, hence a smaller C_p also decreases t_r and increases the frequency.

Supplementary Note 3: The impact of V_{th} fluctuations on spike encoding quality

To emulate V_{th} fluctuations, we superimposed Gaussian noise with a zero mean and varying standard deviations (σ = 0.00 V, 0.01 V, 0.02 V, 0.03 V, 0.05 V, 0.08 V, 0.10 V, 0.13 V and 0.15 V) on the constant V_{th} in our VO_2 SPICE model. At a V_{th} of 3.4 V, this is equivalent to C_v values ranging from 0% to 4.41%. We randomly selected 8 ECG signals and simulated the noisy encoding process ten times for every combination of signal and σ, totaling 720 trials. In each trial, different random noise was regenerated. The mean squared error (MSE) between the original and the reconstructed signal was calculated for each trial.

The average MSE for each C_v and the individual MSE of each trial are plotted in Supplementary Fig. 17a. Also indicated in the figure is the C_v of V_{th_pos} of our VO_2 device. In general, the MSE increases with C_v as a larger fluctuation degrades encoding quality. The distribution of the individual MSE also increases with C_v. Examples of the reconstructed signal, voltage across the VO_2 device and V_{th} for zero, moderate (C_v = 2.35%) and high (C_v = 4.41%) degrees of V_{th} fluctuation are plotted in Supplementary Fig. 17b-d, respectively. By inspecting the signal reconstruction in Supplementary Fig. 17c and comparing its MSE with the individual MSEs for C_v < 0.88% (σ < 0.03 V), we can infer that the proposed encoder based on our VO_2 memristor can perform accurate spike encoding. The tight MSE distribution at low C_v will also contribute to achieving superior repeatability in spike encoding.

Supplementary Note 4. A comparison between different implementations of neurons and asynchronous spike encoders

The proposed VO_2 memristor-based physiological signal processing system has a high area efficiency. To illustrate this, we compared each VO_2 memristor-based module with various other CMOS and memristor implementations.

First, we estimated the area of the VO_2 memristor-based neurons by considering the size of each individual circuit component. After appropriate circuit-level optimizations (Supplementary Table 10), our LIF and ALIF neurons occupy areas of only ~41.3 μm^2 and ~53.4 μm^2, respectively. These figures are three to four orders of magnitude better than those before optimizations. Our ALIF neuron also shows at least ~2.2× area gains when compared to other implementations (Supplementary Table 11). One of the optimizations is to substantially reduce the capacitors as they dominate the area of the neurons. This is possible owing to the ability of VO_2 in switching at ultrafast timescales[20]. Besides, the large resistors in the ALIF neuron should be replaced by transistors operating as current source loads. For the sake of completeness, the speed of the ALIF neurons in terms of the spiking frequency as well as the energy consumption are also given in Supplementary Table 11 (see also Supplementary Fig. 29), wherein we considered an optimized VO_2 memristor with a lower V_{th} and a higher HRS based on ref[13]. We then performed a more comprehensive comparison between ALIF implementations based on a figure of merit (FOM) given by Eq. 4:

$$\mathrm{FOM} = \frac{f}{E \cdot A} \qquad (4)$$

where f, E and A are the spiking frequency, energy per spike and area, respectively. As summarized in Supplementary Table 11, our VO_2 memristor-based ALIF neuron can also achieve the highest FOM after optimizations.

Next, we estimated the area of the VO_2 memristor-based asynchronous spike encoder. In this case, the area is dominated by the op-amps and the capacitors. Here, an optimized VO_2 memristor was again considered[13]. Taking into account the capability of the op-amp in driving the VO_2 load circuit, the area of the op-amp was assumed to be 725 μm^2 as reported in ref[14]. Furthermore, the capacitors were also reduced while maintaining the encoding functionality of the architecture. Along with the size of other individual circuit components (Supplementary Table 12), the area was estimated to be ~2231 μm^2. As summarized in Supplementary Table 13, the optimized VO_2 memristor-based encoder is almost an order of magnitude more compact than other similar encoders.

Therefore, we conclude that with appropriate device and circuit optimizations, a physiological processing system that employs our VO_2 memristor-based encoder and neurons is superior to other CMOS or memristor implementations primarily in terms of area benefits.

Supplementary Reference

1 Indiveri, G., Chicca, E. & Douglas, R. A VLSI array of low-power spiking neurons and bistable synapses with spike-timing dependent plasticity. *IEEE Trans. Neural Netw.* **17**, 211-221 (2006).

2 Indiveri, G. Synaptic Plasticity and Spike-based Computation in VLSI Networks of Integrate-and-Fire Neurons. *Neural Inf. Process. Lett. Rev* **11**, 135-146 (2007).

3 Ferreira, P. M., De Carvalho, N., Klisnick, G. & Benlarbi-Delai, A. Energy efficient fJ/spike LTS e-Neuron using 55-nm node. *2019 32nd Symposium on Integrated Circuits and Systems Design (SBCCI)*, 1-6 (2019).

4 Ahmadi-Farsani, J., *et al.* A CMOS-memristor hybrid system for implementing stochastic binary spike timing-dependent plasticity. *Philos. Trans. A Math. Phys. Eng. Sci.* **380**, 20210018 (2022).

5 Wang, X., *et al.* A novel rram-based adaptive-threshold lif neuron circuit for high recognition accuracy. In: *2018 International Symposium on VLSI Technology, Systems and Application (VLSI-TSA)* (2018).

6 Shaban, A., Bezugam, S. S. & Suri, M. An adaptive threshold neuron for recurrent spiking neural networks with nanodevice hardware implementation. *Nat. Commun.* **12**, 4234 (2021).

7 Sharifshazileh, M., Burelo, K., Sarnthein, J. & Indiveri, G. An electronic neuromorphic system for real-time detection of high frequency oscillations (HFO) in intracranial EEG. *Nat. Commun.* **12**, 3095 (2021).

8 He, Y., *et al.* A 28.2 μC Neuromorphic Sensing System Featuring SNN-based Near-sensor Computation and Event-Driven Body-Channel Communication for Insertable Cardiac Monitoring. In: *2021 IEEE Asian Solid-State Circuits Conference (A-SSCC)* (2021).

9 Liang, W., Pei, H., Cai, Q. & Wang, Y. Scalp EEG epileptogenic zone recognition and localization based on long-term recurrent convolutional network. *Neurocomputing* **396**, 569-576 (2020).

10 Boonyakitanont, P., Lek-uthai, A., Chomtho, K. & Songsiri, J. A comparison of deep neural networks for seizure detection in EEG signals. *bioRxiv*, 702654 (2019).

11 Wei, Z., Zou, J., Zhang, J. & Xu, J. Automatic epileptic EEG detection using convolutional neural network with improvements in time-domain. *Biomed. Signal Process. Control* **53**, 101551 (2019).

12 Wang, X., *et al.* One dimensional convolutional neural networks for seizure onset detection using long-term scalp and intracranial EEG. *Neurocomputing* **459**, 212-222 (2021).

13 Dutta, S., *et al.* Programmable coupled oscillators for synchronized locomotion. *Nat. Commun.* **10**, 3299 (2019).

14 Yosefi, G. The high recycling folded cascode (HRFC): A general enhancement of the recycling folded cascode operational amplifier. *Microelectron. J.* **89**, 70-90 (2019).

15 Jerry, M., *et al.* Stochastic Insulator-to-Metal Phase Transition-Based True Random Number Generator. *IEEE Electron Device Lett.* **39**, 139-142 (2018).

16 Qazilbash, M. M., *et al.* Mott Transition in VO2 Revealed by Infrared Spectroscopy and Nano-Imaging. *Science* **318**, 1750-1753 (2007).

17 Narayan, J. & Bhosle, V. M. Phase transition and critical issues in structure-property correlations of vanadium oxide. *J. Appl. Phys.* **100**, 103524 (2006).

18 Yi, W., *et al.* Biological plausibility and stochasticity in scalable VO2 active memristor neurons. *Nat. Commun.* **9**, 4661 (2018).

19 Nandi, S. K., *et al.* Understanding modes of negative differential resistance in amorphous and polycrystalline vanadium oxides. *J. Appl. Phys.* **128**, 244103 (2020).

20 Frougier, J., *et al.* Phase-Transition-FET exhibiting steep switching slope of 8mV/decade and 36% enhanced ON current. In: *2016 IEEE Symposium on VLSI Technology* (2016).
